# *SLC44A2* Frequency, a New TaqMan Real-Time Polymerase Chain Reaction Method for HNA-3A/3B Genotyping, and a New Application of Droplet Digital PCR

**DOI:** 10.3389/fgene.2022.794285

**Published:** 2022-05-12

**Authors:** Yufeng Wang, Xihui Chen, Qi Chen, Tangdong Chen, Kun Chen, Yuanming Wu, Li Wang

**Affiliations:** ^1^ Department of Biochemistry and Molecular Biology, Fourth Military Medical University, Xi’an, China; ^2^ Shaanxi Provincial Key Laboratory of Clinic Genetics, Fourth Military Medical University, Xi’an, China; ^3^ Medical Genetics, Yan’an University, Yan’an, China; ^4^ Department of Anatomy, Histology and Embryology, K.K. Leung Brain Research Centre, Fourth Military Medical University, Xi’an, China; ^5^ School of Aerospace Medicine, Fourth Military Medical University, Xi’an, China

**Keywords:** *SLC44A2*, HNA-3, TaqMan real-time polymerase chain reaction method, droplet digital PCR, genotype

## Abstract

**Background:** Human neutrophil antigen-3A (HNA-3A) and human neutrophil antigen-3B (HNA-3B) are generated by a single-nucleotide polymorphism (rs2288904, c.461G > A) in exon 7 of the choline transporter-like protein-2 gene (*CTL2*, also known as *SLC44A2*). Antibodies to HNA-3 can be generated following blood transfusion or other factors resulting in exposure to HNA-3 antigens. These antibodies can cause transfusion-related acute lung injury (TRALI) or neonatal alloimmune neutropenia (NAIN). This study describes a sensitive and specific TaqMan real-time polymerase chain reaction (PCR) method to screen for the HNA-3 genotype using specific primers and probes designed to detect allelic polymorphisms. Considering the high sensitivity and accuracy of droplet digital PCR (ddPCR) in the identification of the rare SLC44A2*2 allele, we used this technique to identify blood donors with the rare HNA-3B antigen and calculate the allele frequency of *SLC44A2* in mixed populations with different proportions.

**Methods:** DNA samples purified from 208 donors in northwest China were subjected to TaqMan real-time PCR to detect allelic polymorphisms in *SLC44A2*. The results were confirmed by Sanger sequencing. The rare HNA-3B antigen was detected by ddPCR. *SLC44A2* frequency was determined by two-channel ddPCR.

**Results:** The genotypes of all DNA samples were detected by the TaqMan real-time PCR using specific probes for HNA-3, and the results were consistent with the Sanger sequencing results in respect to the HNA-3A and HNA-3B polymorphisms. The allele frequencies of SLC44A2*1 and SLC44A2*2 in the 208 donors in northwest China were 64.9% (95% confidence interval [CI], 59%–70.8%) and 35.1% (95% CI, 29.2%–41%), respectively. The ratio of SLC44A2*2 alleles was accurately detected in all blood pools by ddPCR but not by TaqMan real-time PCR. This allowed for the *SLC44A2* frequency in the population to be accurately inferred.

**Conclusion:** This new method of detecting *SLC44A2* alleles was highly sensitive and specific, as confirmed by Sanger sequencing. ddPCR using the designed probes resulted in successful detection of the rare HNA-3B antigen. Furthermore, we successfully detected the rare HNA-3B antigen and inferred the *SLC44A2* frequency by ddPCR using the probes that we designed.

## Introduction

Population genetics is the study of gene distribution, gene frequency and genotype frequency maintenance, and change in a population (Okazaki et al., 2021). Population genetics reveals the genetic relationship between gene and genotype frequencies of a random mating population by the Hardy–Weinberg Principle, but gene frequency is affected by various factors in practice, such as mutation, natural selection, genetic drift, in-migration, and out-migration (Salanti et al., 2005). Therefore, the design of a simple and accurate method for genotype and gene frequency statistics is of great importance. Blood group genes are one of the key markers of population genetics, and the distribution frequency of blood group genes is an important issue in population genetics (Sakaue et al., 2021). There is a strong link between blood group genes and the expression of blood group antigens (Tournamille, 2018). Owing to genetic drift and the founder effect, the frequency of blood group genes varies from region to region (Belsito et al., 2017). Accurate classification of blood groups is of great importance in the study of clinical blood transfusion, organ transplantation, human kinship, and ethnic migration.

In 2019, the International Society of Blood Transfusion (ISBT) announced a newly discovered red blood cell blood group antigen, the CTL2 blood group system antigen, which is widely expressed in blood cells and tissues (Flesch et al., 2013). The CTL2 blood group antigen is encoded by the choline transporter-like protein 2 gene, also known as the *SLC44A2*, which is located on human chromosome 19p13.1 (Nair et al., 2016). A single-nucleotide polymorphism in exon 7 of *CTL2* (*SLC44A2*) results in a base substitution at SNP rs2288904 (c.461G > A) and an amino acid substitution in the encoded protein, giving rise to the HNA-3A and HNA-3B antigens. HNA-3 antigen has been identified as an etiological factor in various immune system diseases, such as neonatal alloimmune neutropenia (NAIN) and transfusion-related acute lung injury (TRALI) (Valle Del Barrio et al., 2021). NAIN is associated with an incompatibility between the unborn baby and the maternal immune system (Arneth, 2020), whereas TRALI is a type of acute respiratory distress that occurs in large numbers of patients within 6 h of blood transfusion (Semple et al., 2019). Antibodies to HNA-3A can be produced following exposure to HNA-3A antigen, due to alloimmunity between the pregnant woman and the fetus or to blood transfusion (Bux, 2008). The incidence of neutropenia in neonates with HNA-3 is 14.8% (Abbas et al., 2021). HNA-3 is also responsible for 0.1% of the incidence of TRALI (Roubinian, 2018).

The frequency of *SLC44A2* alleles encoding HNA-3 antigens has been found to vary in different populations (Huvard et al., 2012). The allele frequencies of SLC44A2*1 (HNA-3A antigen-coding allele) and SLC44A2*2 (HNA-3B antigen-coding allele) are 76.8% and 23.2%, respectively, in European blood donors of Caucasian ancestry (Cardoso et al., 2013); 65.4% and 34.6%, respectively, in a Japanese population (Matsuhashi et al., 2012); and approximately 70% and 30%, respectively, in a Chinese population (Ou et al., 2018). In addition, the allele frequency of SLC44A2*2 varies greatly between different regions. For instance, the allele frequency of SLC44A2*2 ranges from 0% to 1.9% in sub-Saharan Africans but only ranges from 0% to 0.05% in Amerindians and Zambians. Compared to any Western population, the chance of being exposed to an alloimmunization risk against HNA-3A is two times greater in Asian populations (Ou et al., 2018). As anti-HNA-3A is more common in Asians, detection of HNA antibodies and the *SLC44A2* genotype in blood donors is thought to improve blood transfusion safety in Asian populations. Therefore, to improve the diagnosis and prevention of clinical diseases mediated by the neutrophil antibodies, there is a clear need for a highly efficient genotyping method to detect *SLC44A2* variations.

Various methods have been reported for rapid *SLC44A2* allele frequency determination in a population, including polymerase chain reaction (PCR)-restriction fragment length polymorphism (RFLP) (Lopes et al., 2014), TaqMan real-time PCR (Nielsen et al., 2012), multiplex PCR (Nathalang et al., 2015), and the use of sequence-specific primers (SSPs) (Intharanut et al., 2019). Owing to its simplicity and low cost, PCR with SSPs followed by agarose gel electrophoresis is widely used for this purpose. However, the use of SSPs is characterized by a high false-positive rate, and it cannot accurately classify point mutations (Barbano et al., 2015). In recent years, because of its high sensitivity and accuracy, TaqMan real-time PCR has been increasingly employed for the determination of allele frequencies (Nielsen et al., 2012). Several commercial kits are currently available to detect *SLC44A2* genotypes, but the primer and probe sequences have not been made publicly available. This hence necessitates reliance on commercial kits, which greatly increases the cost of this testing. In addition, although the TaqMan PCR method can accurately classify *SLC44A2* variants, it cannot detect rare alleles in mixed samples and perform absolute quantification of samples. When testing a large number of samples, therefore, there is still a need to obtain and test individual samples of DNA. This greatly increases the costs of reagents, consumables, and human resources. Therefore, TaqMan PCR is not suitable for investigation of gene distribution frequencies in large populations. There is, hence, a pressing need to develop an efficient, simple, and low-cost technique to investigate the frequency of the *SLC44A2* allele in populations.

Droplet digital PCR (ddPCR) is a quantitative determination method based on the Poisson distribution (Morley, 2014). With this method, the target sample is divided into numerous droplets, each of which contains only a single target DNA. Each of these target DNAs is PCR amplified, with the resulting fluorescence signal enabling calculation of copy numbers in the target samples using the Poisson distribution while analyzing the properties of samples (Hindson et al., 2011). Because the detection of rare entities by ddPCR is a highly sensitive alternative method, use of this method to search for rare alleles in blood donors may enable screening for rare erythrocyte phenotypes. In addition, ddPCR can also be employed with multiple samples at the same time, thereby greatly reducing the difficulty and cost of testing while still ensuring efficiency (Dezan et al., 2020). Therefore, ddPCR is widely used in mutation screening of populations (Boone et al., 2020).

In this study, we devised a ddPCR assay based on the TaqMan probe method and proved its effectiveness. We generated blood pools of a large number of HNA-3*A/*A samples mixed with several HNA-3*A/*B samples. We were able to detect the HNA-3B allele in these blood pools using ddPCR, thereby demonstrating the sensitivity of this method and establishing its suitability for the detection of rare HNA-3B antigens in the population. In addition, we generated blood pools with different HNA-3A and HNA-3B gene ratios to further characterize the determination of the *SLC44A2* frequency in the population. The effective detection of these two blood pools demonstrates the effectiveness of this method for detecting rare genes and for determining gene frequency. In addition, the establishment of the suitability of this method represents a key step forward for *SLC44A2* screening as well as NAIN and TRALI prevention.

## Materials and Methods

### Donor Population Recruitment

208 donors mainly from northwest of China were recruited in our study to calculate allele frequency of *SLC44A2*. 2 ml fresh blood of each donor was collected by using EDTA tubes. The genomic DNA was extracted from each blood samples by using Ezup Column Blood Genomic DNA Purification Kit (Sangon Biotech) according to the instruction. For this study, blood donors have been given informed consent. Meanwhile This study was approved by the Ethics Committee of Fourth Military Medical University and complies with the declaration of Helsinki.

### HNA-3 Genotyping by TaqMan Real-Time PCR

A TaqMan real-time PCR was established for genotyping of DNA samples. A new assay was standardized for the evaluation of this SNPs by real-time PCR in 25 μL reactions using the primers and probes described in Table 1 (Sangon Biotech). The assays used 2 × Premix Ex Taq (Probe qPCR), 50 × ROX Reference Dye II, (Premix Ex Taq™ (Probe qPCR); TAKARA) 30 ng of genomic DNA, the final concentration of primer and probe is 0.4 μM. Both reactions were performed in a real-time PCR system, ABI 7500 Fast (Applied Biosystems), under the following conditions: a pre-amplification step of 60°C for 1 min and 95°C for 10 min, followed by 40 cycles at 95°C for 15 s and 60°C for 1 min, as well as a post-amplification step of 60°C for 1 min.

**TABLE 1:** Primers and probes used for HNA-3A/HNA-3B (*CTL2* system) genotyping.

System	Primers and probes	Sequences
CTL2	CTL2 F4	5′-CGC​ATG​CAC​TTA​TTC​ACG​GG-3′
	CTL2 R4	5′-GGC​ACA​GTG​AGG​ATG​AGG​AC-3′
	CTL2 3A	5′6-FAM-CCATCTCGAAGCACCT-3′MGB
	CTL2 3B	5′6-VIC-CCATCTTGAAGCACCT-3′MGB

### HNA-3 Nucleotide Sequencing

Each sample was amplified and sequenced to check against the PCR results. Each sample was subjected to amplification reaction covering HNA-3 SNP region of 30 μL: ddH_2_O 9 μL, 1X buffer 15 μL (2×Taq PCR Mix, RUNDE), the primers included CTL ID FP (CAC​GTA​CCT​GAA​TGC​TCG​C) and CTL ID RP (GAG​CAG​AGG​ATG​GCA​CCA​GT) primer (10 μM) 1.5 μL (Sangon Biotech). Thermal Circulator (ProFlex PCR System, Applied Biosystems by Life Technologies) at 95°C for 10 min; 40 cycles 94°C for 30 s, 58°C for 1 min, 72°C for 30 s, the last step is 98°C for 10 min, 4°C ∞. Sequencing was performed after PCR amplification.

### Fifty Donor Pool Organization for Detecting Rare HNA-3B Antigen

For validation purpose, nucleic acid of 50 donors with known HNA-3A and HNA-3B genotype were organized. We collected 50 HNA-3*A/*A samples, which were treated as “control” pools. The other samples were organized as follows: 1) Pool 1.1 contained 49 HNA-3*A/*A donors and 1 HNA-3*A/*B donor; 2) Pool 1.2 contained 48 HNA-3*A/*A donors and 2 HNA-3*A/*B donors; 3) Pool 1.3 contained 47 HNA-3*A/*A donors and 3 HNA-3*A/*B donors; 4) Pool 1.4 contained 46 HNA-3*A/*A donors and 4 HNA-3*A/*B donors; 5) and Pool 1.5 contained 45 HNA-3*A/*A donors and 5 HNA-3*A/*B donors.

### Fifty Donor Pool Organization for Inferring *SLC44A2* Gene Frequency

There are 208 donors whose genotypes are known. We collected 50 HNA-3*A/*A samples, which were treated as “control” pools. The other samples were organized as follows: 1) Pool 2.1a contained 40 HNA-3*A/*A donors and 10 HNA-3*A/*B donor; 2) Pool 2.1b contained 45 HNA-3*A/*A donors and 5 HNA-3*B/*B donors; 3) Pool 2.2a contained 30 HNA-3*A/*A donors and 20 HNA-3*A/*B donors; 4) Pool 2.2b contained 40 HNA-3*A/*A donors and 10 HNA-3*B/*B donors; 5) Pool 2.3a contained 20 HNA-3*A/*A donors and 30 HNA-3*A/*B donors; 6) Pool 2.3b contained 35 HNA-3*A/*A donors and 15 HNA-3*B/*B donors; 7) Pool 2.4a contained 10 HNA-3*A/*A donors and 40 HNA-3*A/*B donors; 8) Pool 2.4b contained 30 HNA-3*A/*A donors and 20 HNA-3*B/*B donors; 9) Pool 2.5a contained 50 HNA-3*A/*B donors; 10) and Pool 2.5b contained 25 HNA-3*A/*A donors and 25 HNA-3*B/*B donors.

### Droplet Digital PCR Assay

Droplet digital PCR probe and primer sequences are same as Taqman probes. For the ddPCR experiment, a master mix was created by adding: 10 μL of ddPCR supermix for probes (Bio-Rad, United States), 2 μL of primers-probe solution (Sangon Biotech), 7 μL of ddH_2_O and 1 μL of DNA of the 50-donor pool, reaching a final reaction volume of 20 μL. Twenty microliters of this solution were added to 8 compartments of the Droplet Generator DG8 Cartridge (Bio-Rad, United States) and droplets were generated. The entire droplet emulsion volume was then loaded into a 96-well PCR plate (Bio-Rad, United States). The loaded PCR plate was sealed with heat in the P X 1 PCR Plate Sealer (Bio-Rad, United States) and placed in a GeneAmp PCR System 9700 (Applied Biosystems) with the following program: incubation for 10 min at 95°C, then 40 cycles of 94°C for 30 s, 60°C for 1 min, and 98°C for 10 min. After PCR amplification, the droplets were analyzed in a QX200 droplet reader Digital PCR System (Bio-Rad, United States), and the quantification of PCR targets was analyzed using QuantaSoft™ software version 1.7.4.0917. The results were reported as the number of copies per microliter (copies/μl).

### Statistical Analysis

Data were analyzed using GraphPad Prism 8 software (GraphPad Software, San Diego, CA) and presented as mean and 95% confidence intervals (CI) based on the Poisson distribution.

## Results

### Sequence Analysis

**FIGURE 1:**
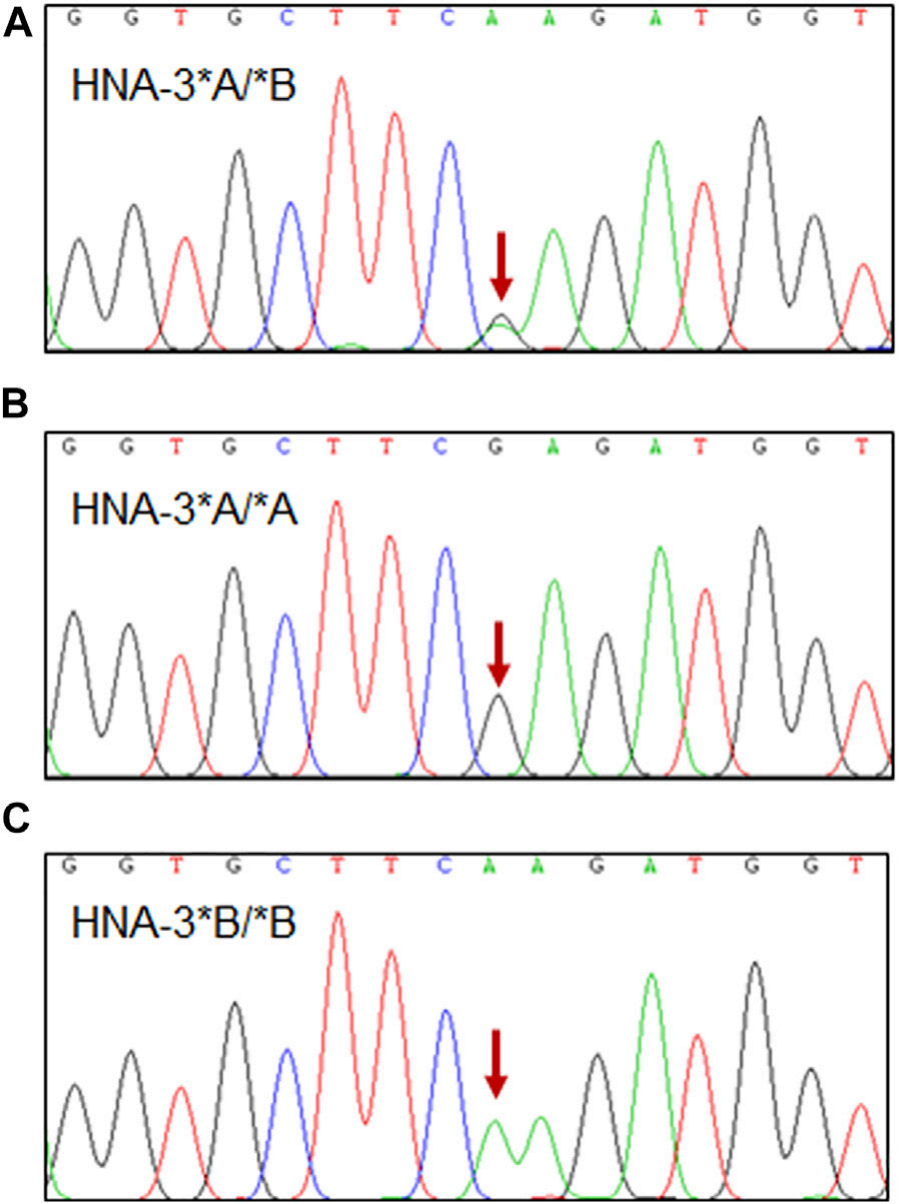
Sanger sequencing results of three representative HNA-3 genotype. **(A)** HNA-3*A/*B genotype, the red arrow indicates the heterozygous G/A genotype at position c.461 in *SLC44A2* gene. **(B)** HNA-3*A/*A genotype, the red arrow indicates the homozygous G/G genotype at position c.461 in *SLC44A2* gene. **(C)** HNA-3*B/*B genotype, the red arrow indicates the homozygous A/A genotype at position c.461 in *SLC44A2* gene.

Primers were designed to amplify a specific 300 bp fragment of an *SLC44A2* region that includes nucleotide position 461. Sanger sequencing of the involved nucleotide region was performed on all 208 blood samples. (Figure 1). The results show that the TaqMan real-time analytical system was 100% specific for identifying HNA-3 polymorphisms in these 208 samples.

### TaqMan Real-Time Polymerase Chain Reaction Analysis

**FIGURE 2:**
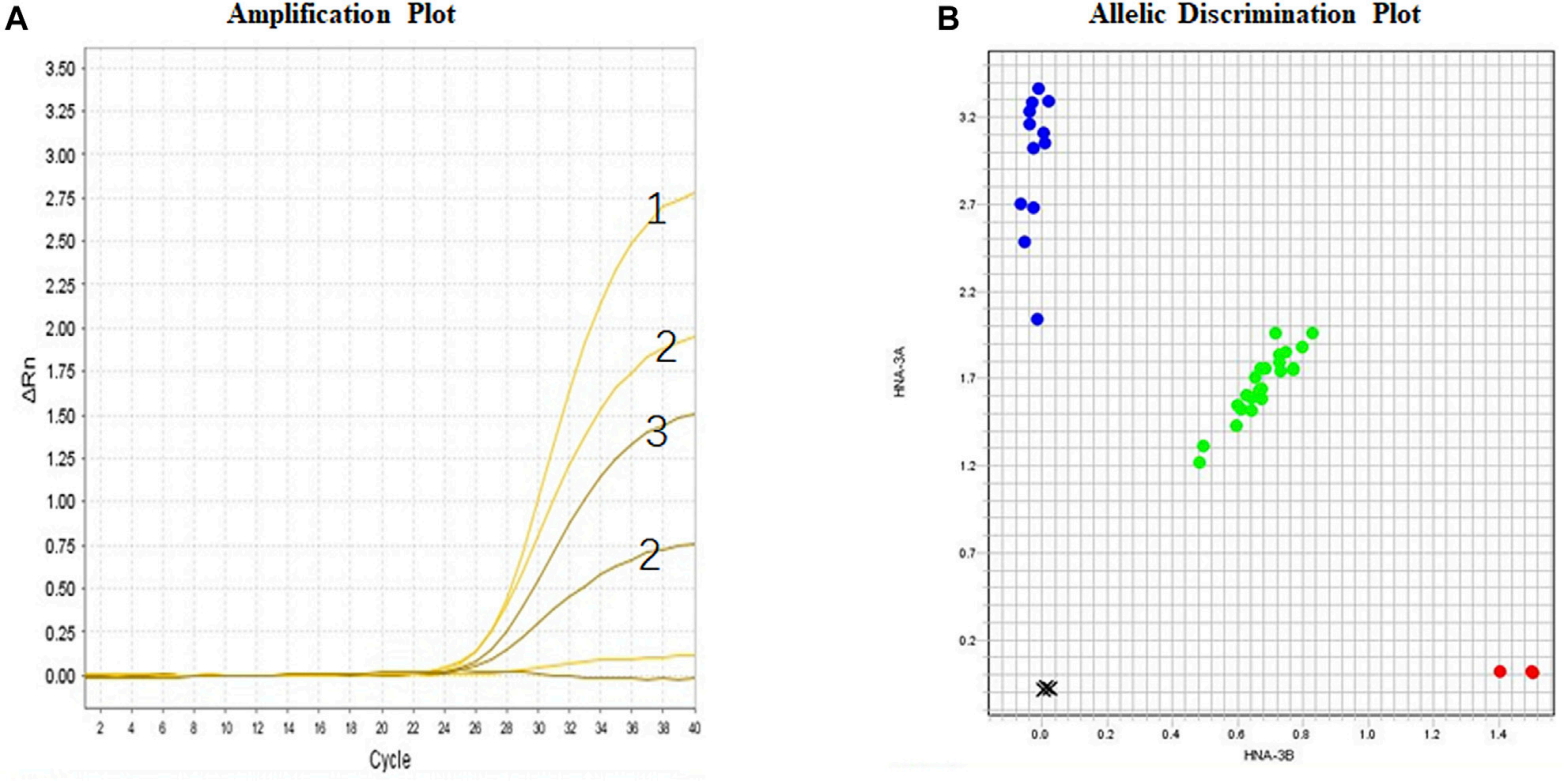
Real-time PCR for *SLC44A2* genotyping (CTL2 system). **(A)** Amplification Plot: HNA-3B-VIC channel; HNA- 3A-FAM channel; Sample No. 1 is SLC44A2*1/1; Sample No. 2 is SLC44A2*1/2; Sample No. 3 is SLC44A2*2/2; **(B)** Allelic discrimination plot for individual samples using purified DNA.

The results of TaqMan genotyping and allele discrimination of individual samples are shown in Figure 2. Individuals with known SLC44A2*1/1, SLC44A2*1/2, or SLC44A2*2/2 genotypes can be clearly identified by the amplification plot. Further, the genotypes of all DNA samples obtained by TaqMan real-time PCR were consistent with the Sanger sequencing results in respect to the HNA-3A and HNA-3B polymorphisms. Further analysis of 208 blood donors in the northwest Chinese population showed that 26 (12.5%) were homozygous for SLC44A2*2 (Table 2), and they were, thus, expected to express only HNA-3B. Eighty-eight (42.3%) individuals were homozygous for SLC44A2*1, and they were, thus, expected to express only HNA-3A. Ninety-four individuals (45.2%) were heterozygous for SLC44A2*1 and SLC44A2*2, and they were, thus, expected to express both HNA-3A and HNA-3B.

**TABLE 2:** Genotype frequencies of *SLC44A2-*expressing HNA-3 variants.

SLC44A2genotype	Predicted HNA-3 expression	Donors(n)	Population frequency	
			Mean*	95% CI*
SLC44A2*1, SLC44A2*1	HNA-3*A/*A	88	42.3%	36.4%–48.2%
SLC44A2*1, SLC44A2*2	HNA-3*A/*B	94	45.2%	39.3%–51.1%
SLC44A2*2, SLC44A2*2	HNA-3*B/*B	26	12.5%	8.6%–16.4%
Total		208		

*95% confidence interval (CI) calculated by Poisson distribution.

### Droplet Digital PCR Analysis for the Detection of Rare HNA-3B Antigen

**FIGURE 3:**
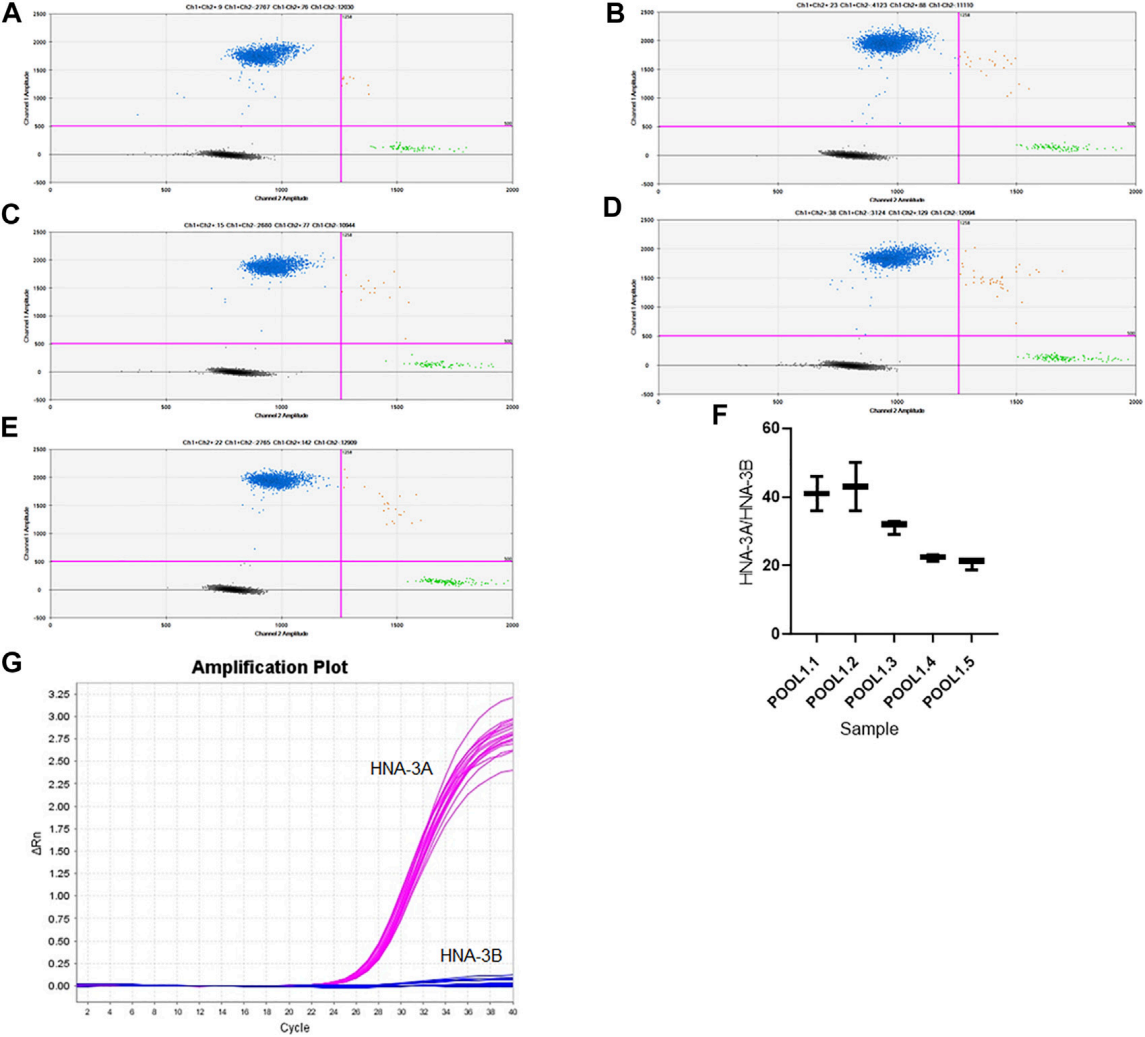
Visualization of the 2D projection of the droplets from the QuantaSoft software. The vertical axis shows the Channel 1 amplitude and horizontal axis shows the Channel 2 amplitude. **(A)** Pool 1 included one HNA-3*A/*B and 49 HNA-3*A/*A donors; **(**B) Pool 2 included two HNA-3*A/*B and 48 HNA-3*A/*A donors; **(C)** Pool 3 included 3 HNA-3*A/*B and 47 HNA-3*A/*A donors; **(D)** Pool 4 included 4 HNA-3*A/*B and 46 HNA-3*A/*A donors; **(E)** Pool 5 included 5 HNA-3*A/*B and 45 HNA-3*A/*A donors; **(F)** Concentration ratios of channel 1 (FAM) to channel 2 (VIC); **(G)** Amplification Plot of HNA-3A (pink) and HNA- 3B (blue) by TaqMan real-time PCR.

HNA-3B antigen produced by the SLC44A2*2 allele is very rare in most populations. ddPCR could potentially be a suitable method to screen for blood donors with the HNA-3B phenotype in pools of samples by detection of the rare SLC44A2*2 allele. As shown by our results, the concentration of SLC44A2*1 allele was accurately determined in all studied pools by using ddPCR (pools 1.1–1.5). Visualization of the 2D projection of the droplets is displayed in Figures 3A–E. Channel 1 represents the copy number and concentration of the SLC44A2*1 allele, whereas channel 2 represents the copy number and concentration of the SLC44A2*2 allele. The mean ± SD ratios of the concentrations of channel 1 (FAM) to channel 2 (VIC) for pools 1.1–1.5 were 41 ± 2.887, 43.00 ± 4.041, 31.33 ± 1.202, 22.20 ± 0.5686, and 20.57 ± 0.9838, respectively (Figure 3F). An increase in the number of blood donors with the SLC44A2*2 allele in the blood pool was accompanied by a significant reduction in the concentration ratio of channel 1 to channel 2. Therefore, the proportion of the SLC44A2*2 allele in each blood pool could be roughly estimated by the ratio of the two channels.

We also sought to detect the rare SLC44A2*2 allele in pools 1.1–1.5 by using TaqMan real-time PCR. However, the results of the TaqMan real-time PCR showed no amplification plot for the SLC44A2*2 allele, indicating that TaqMan real-time PCR is not sufficiently sensitive to detect rare alleles (Figure 3G). These results further demonstrate that ddPCR has a clear advantage compared with TaqMan real-time PCR owing to its high sensitivity for detection of rare HNA-3B antigen in multiple blood samples.

### Droplet Digital PCR Analysis for Inferring *SLC44A2* Frequency

**FIGURE 4:**
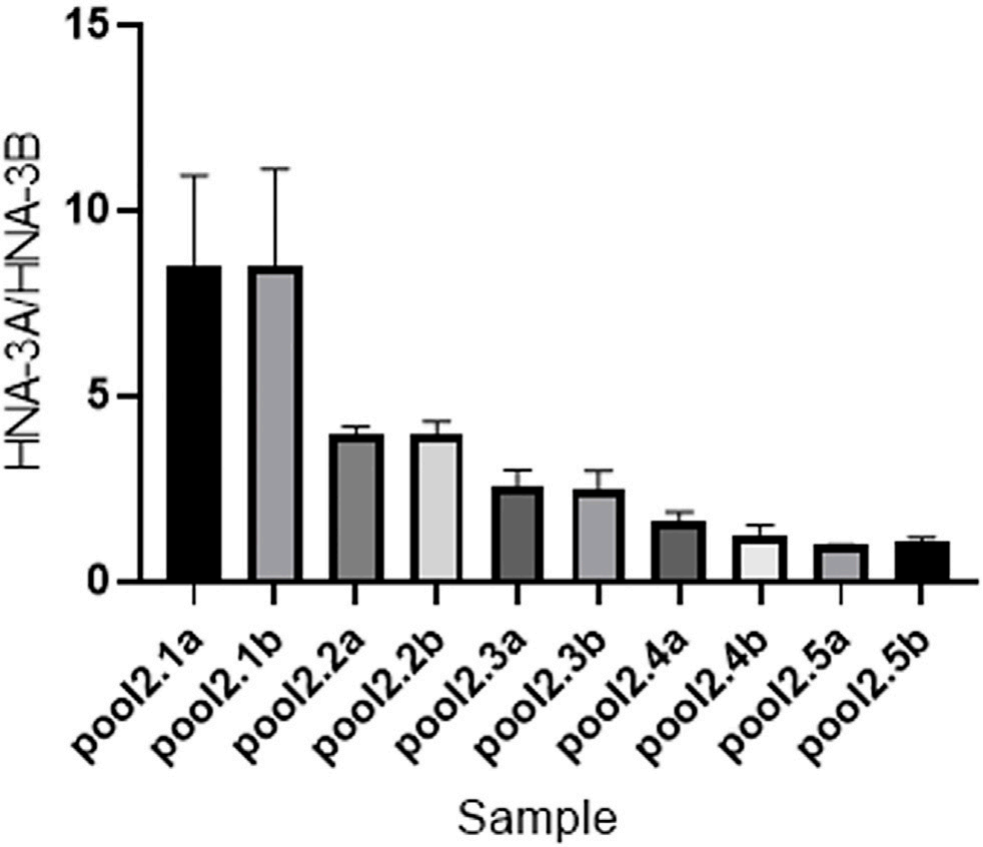
Concentration ratios of channel 1 (FAM, HNA-3A) to channel 2 (VIC, HNA-3B).

The result of ddPCR was consistent with the actual frequency of *SLC44A2*. Statistical results of digital PCR are shown in Figure 4. Channel 1 represents the copy number and concentration of the SLC44A2*1 allele, whereas channel 2 represents the copy number and concentration of the SLC44A2*2 allele. The mean ± SD ratios of the concentrations of channel 1 (FAM) to channel 2 (VIC) for pools 2.1a–2.5b were 8.500 ± 0.9849, 8.500 ± 1.054, 3.983 ± 0.08083, 3.947 ± 0.1450, 2.567 ± 0.1704, 2.507 ± 0.1901, 1.607 ± 0.1060, 1.250 ± 0.1054, 0.9533 ± 0.0115, and 1.063 ± 0.04726, respectively. These results show that the sensitivity and accuracy of ddPCR analysis for determining allele frequency are not affected by the genotypes of the individuals in the pool.

## Discussion

The present study was designed to determine the allele frequency of the HNA-3 epitope encoded by *SLC44A2* and to identify the rare HNA-3B antigen in the selected population using droplet digital PCR.

Screening blood donors for anti-HNA-3A alloantibodies is of considerable importance for safe blood transfusion. Until recently, this was not feasible at most donor centers owing to the need for complex serotyping with rare antisera. The real-time PCR assay described in this study is a simple, fast, sensitive, and specific method for screening all blood donors.

Allele-specific PCR is a widely used PCR method for detecting DNA sequence variants, by designing the forward primer with the mutation site at the 3′ end. However, this method tends to provide spurious results. For example, the absence of the rare SLC44A2*1 variant can result in the donor being incorrectly labeled as homozygous for SLC44A2*2, a genotype present in approximately 0.07%–0.4% of all donors, depending on the allele frequency. By contrast, TaqMan real-time PCR can accurately determine HNA-3A and HNA-3B antigen types.

HNA-3B is a rare antigen among Zambians and Amerindians, necessitating screening of these populations for blood donors with this rare antigen. To reduce the cost of DNA extraction, blood samples were pooled from 50 donors, and the presence of the gene encoding the rare antigen HNA-3B was analyzed in DNA extracted from these pools. In addition to identifying the presence of genes encoding these rare antigens, this ddPCR method was able to provide a rough estimate of the number of samples in each pool that contained the rare alleles.

This method can be applied to immunohematology and fetal medicine. Strategies using ddPCR are more sensitive than those using qPCR in identifying donors with rare alleles, thereby enabling expansion of the number of donors in each donor pool. This method can also be used to search for other rare erythrocyte antigens with low frequency, but only if the method can identify a pair of rare alleles that predict rare phenotypes when homozygous. Additionally, because multiple samples are tested simultaneously and accurately, ddPCR can be used to measure the frequency of variants in population genetics in a more cost-effective manner. In this study, the established ddPCR method was able to process at least 50 samples in one test simultaneously, at an average cost of $2.69 per sample ($134.5 per test). By contrast, the average cost of the TaqMan real-time PCR method is higher, at approximately $51.92 per sample (three replicates).

This study is also the first to describe the application of ddPCR for determination of gene frequency. The method is sensitive and accurate for determination of *SLC44A2* frequency, which implies expanded application in other blood groups with single-nucleotide mutations. Additionally, compared with the traditional method whereby the genotype should be examined for each donor, the use of blood pools can significantly improve the detection efficiency when estimating gene frequencies in a large population.

## Conclusion

This study described a sensitive and specific screening method for HNA-3 genotyping. The high sensitivity and accuracy of this technique in the identification of rare SLC44A2*2 alleles make this method feasible for screening donor blood for the rare HNA-3B antigenic phenotype. This ddPCR method represents an alternative approach to screening blood donors with rare alleles and may be applicable in screening for other rare red blood cell phenotypes; furthermore, it allows for inferring the *SLC44A2* frequency.

## Data Availability

The original contributions presented in the study are included in the article/Supplementary Material, further inquiries can be directed to the corresponding authors.
